# Sustainable 3D Scaffolds Based on β-Chitin and Collagen I for Wound Dressing Applications

**DOI:** 10.3390/polym17020140

**Published:** 2025-01-08

**Authors:** Marianna Barbalinardo, Giuseppe Falini, Devis Montroni

**Affiliations:** 1 National Research Council (CNR), Institute for Nanostructured Materials (ISMN), Via P. Gobetti 101, 40129 Bologna, Italy; 2 Dipartimento di Chimica “G. Ciamician”, Alma Mater Studiorum−Università di Bologna, Via F. Selmi 2, 40126 Bologna, Italy

**Keywords:** chitin, collagen, foam, sponge, adhesion, green chemistry, wound healing, wound dressing

## Abstract

The development of greener substitutes for plastics is gaining massive importance in today’s society. This also involves the medical field, where disposable materials are used to grant sterility. Here, a novel protocol using only a water-based solvent for the preparation of bio-based composite foams of actual β-chitin and collagen type I is presented. The influence of the ratio of this chitin polymorph to the collagen on the final material is then studied. The samples with 50:50 and 75:25 ratios produce promising results, such as remarkable water absorption (up to 7000 wt.%), exposed surface (up to 7 m^2^·g^−1^), and total pore volume (over 80 vol.%). The materials are also tested using wet mechanical compression, exhibiting a Young’s modulus and tenacity (both calculated between 2% and 25% of deformation) of up to 20 Pa and 9 kPa, respectively. Fibroblasts, keratinocytes, and osteoblasts are grown on these scaffolds. The viability of fibroblasts and keratinocytes is observed for 72 h, whereas the viability of osteoblasts is observed for up to 21 days. Under the two conditions mentioned, cell activity and adhesion work even better than under its counterpart condition of pure collagen. In conclusion, these materials are promising candidates for sustainable regenerative medicine scaffolds or, specifically, as biodegradable wound dressings.

## 1. Introduction

In the last few decades, the increase in environmental pollution and the amount of dumped plastic material have led to a necessary shift from petroleum-based materials to more eco-friendly alternatives. This change is affecting all industrial fields, including the medical one, especially when disposable materials are used [1,2]. In fact, it is estimated that US hospitals produce about 5.9 million tons of waste annually, mostly disposable materials used on patients [1]. Among these waste materials, wound dressing constitutes a relevant portion. As a reference, the UK’s National Health Service managed an estimated 2.2 million patients with a wound during 2012/2013 (4–5% of the adult population), and about 50% of these wounds were ulcers or burns that required absorbing dressings [3]. Most of these absorbing dressings consist of soft polymers, foams, or hydrogels [4,5,6,7] produced using non-biodegradable materials such as polyurethane, polyvinyl pyrrolidone, or polyacrylamide [8,9]. Considering an average weight of a dressing of about 60 g, this means that, in one year in the UK, about 660 tons of dressing waste is produced. Observing these numbers, it becomes clear that a transition away from these dressings to greener materials is required, while, of course, retaining their efficiency.

This green shift does not only involve the material itself but also the methodology used to obtain it, resulting in the avoidance of toxic organic solvents or chemical reagents, which is aligned with a decrease in the material’s potential toxicity due to chemical traces.

Collagen is one of the most abundant proteins in the animal kingdom [10,11,12,13,14,15,16,17,18]. In humans, it is found in connective and bone tissues. As an example, collagen type I constitutes about 80 wt.% of the dry weight of the skin’s extracellular matrix and also 55–75 wt.% of tendons [19,20,21]. This protein has been widely applied in material sciences, exploiting its remarkable biocompatibility for medical applications [17,22,23,24]. Collagen is also found in a wide range of animal wastes, from the food (i.e., tendons, skin, intestines, and bones) to the clothing industry (i.e., hides) [25,26,27,28]. Nowadays, many different studies have targeted new methods to treat these wastes to recover collagen [26,27,28].

Chitin, on the other hand, is a polysaccharide formed by N-acetyl-glucosamine monomers [29,30,31,32]. It is the most diffused biopolymer among the existing species and is commonly found in mechanically resistant and supportive structures [33,34,35,36,37,38,39]. Chitin also represents the second most abundant biopolymer on earth, after cellulose, and can be easily purified from food waste [40]. Although cellulose is widely exploited (also used in gauzes for cut or surgical wound dressings), chitin has very few commercial uses. Chitin’s biocompatibility, associated with its remarkable wet mechanical properties, promotes its use in medical applications, from drug delivery to wound healing and regenerative medicine [41,42,43,44,45,46]. As an example, labeling studies on rabbits by Ge et al. (2004) showed how a chitinous matrix not only promoted osteoblast proliferation but also promoted the ingrowth of the surrounding tissues [43]. Another interesting effect observed in chitinous materials is the formation of granulation tissue with angiogenesis [47]. It has also been reported that chitin and its metabolites induce fibroblasts to release interleukin-8, which is involved in the migration and proliferation of fibroblasts and vascular endothelial cells [41,48,49]. These properties make chitin an interesting candidate for applications in the biomedical field. Despite that, chitin’s positive biological effects arise only when combined with other components. In fact, compared to collagen, chitin by itself does not induce high cell adhesion and proliferation rates, even though it appears to enhance other molecules’ effects. For this reason, different blends with other materials have been studied [49,50,51,52,53,54]. As an example, despite good results being obtained using a collagen-based material, Li et al. (2006) reported how their implant on a goat led to perfect recovery only when reinforced with chitin [49]. The same group reported an increase in the growth rate of mesenchymal stem cells when testing a different composite based on chitin and collagen [54].

To date, a diverse body of literature exists on chitin/collagen composites. In these studies, chitin solutions or dispersions are prepared using either of the following:

- Organic solvents as a mixture of dimethylacetamide (DMAc) and LiCl 5% [50], hexafluoroisopropanol (HFIP) [55,56], or a mixture of CaCl_2_ and methanol [57,58,59];
- Highly concentrated (2–5 M) NaOH (or KOH) and urea (≈0.6 M) mixtures [60,61]. These highly alkaline mixtures are applied for long reaction times (days) and likely induce the degradation of the polymer (i.e., deacetylation or chain shortening), which is generally not tested. In addition, energy- and time-consuming repeated freeze/thawing cycles are required to obtain a proper chitin dispersion.

These dissolution processes also imply the complete solubilization of the polymer with a consequent conversion of β-chitin into α-chitin [62]. To our knowledge, no prior study has ever reported an actual β-chitin/collagen sponge-like material. Alternatively, α-chitin nanocrystals, or nanofibrils, are often used as the chitin source [49,54,63,64]. The production of these nanomaterials requires the use of harsh acid or alkaline conditions, or oxidizing agents frequently combined with high temperatures, and long digestion times, often leading to low production yield [65,66,67]. Here, the protocol proposed was able to deliver a dispersion of β-chitin in 10 min at room temperature with close to 100% yield using a solvent milder than commercial vinegar. This method places this study on a completely different level compared to studies using nanomaterials.

The chitin dispersion obtained was used to prepare composite foams, combining the biological effect of collagen I and the enhancing biological effect of chitin along with its mechanical resistance. In addition, the use of the β-chitin polymorph led to a more hydratable material compared to the α-chitin polymorph [68,69].

The aim of this project is the green synthesis of biodegradable and/or bioresorbable bio-based scaffolds for medical applications, prepared without the use of toxic reagents and only employing water-based solvents and waste material from edible animal tissues (chitin and collagen). Successively, we explored the influence of the β-chitin/collagen ratio from both the material and biological point of view. This represents new knowledge on the topic since the interaction of collagen I with the β-chitin polymorph has never been discussed in 3D materials.

We believe this innovative green manufacturing process to produce chitin and collagen foam composites, and the knowledge derived from their characterization will provide greener materials for biomedical applications, especially as wound dressing for exudating wounds.

## 2. Materials and Methods

### 2.1. Materials

All reagents and solvents were purchased from Merck and utilized without any further purification. A type I collagen from bovine Achille’s tendon was used in this study. Squid pens from Loligo vulgaris were collected from a local market. Once hydrated, the lateral blades were isolated, cleaned with distilled water and ethanol 70 vol.%, rinsed again with distilled water to remove the ethanol, and then stored dry.

### 2.2. β-Chitin Purification from the Squid Pen

**Figure 1:**
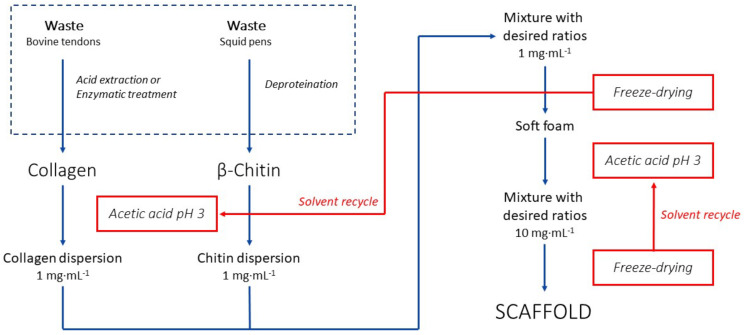
Schematic representation of the entire synthetic process of the scaffolds. A dashed line shows a potential route of synthesis of collagen from animal tissue wastes (i.e., tendons), which is not explored in this work. Red lines indicate how the solvent can be recovered during freeze-drying and reused to repeat the scaffold synthesis.

β-chitin was purified from the squid pen of *L. vulgaris*, a natural composite of chitin and proteins [70], by alkaline deproteination [29,33,68,71]. The purification was performed using a standard protocol, and its optimization and environmental improvement were not part of this study. This was performed by inserting 2.5 g of washed squid pens in 100 mL of a boiling 1 M NaOH solution and stirring for 1 h to cleave the peptide bond of proteins. After that, the solution was changed with a fresh 1 M NaOH solution and refluxed for an additional 1 h. The obtained chitin films were washed at room temperature first with a 1 M NaOH solution and then with distilled water until the washing solution had a neutral pH. The chitin films were stored dry in a desiccator. This step is reported in Figure 1 as deproteination. At the end of this purification process, the chitin obtained was still in the β-chitin polymorph, with a degree of acetylation of 89% (obtained by solid-state NMR) and a molecular weight over 500 kDa (obtained using dynamic light scattering). The purity of the biopolymer was verified using NMR, FTIR, and UV spectroscopy (for tryptophan absorption); no signals other than chitin signals were detected. These results are reported in Montroni et al. (2019 and 2021) [29,33].

### 2.3. Preparation of the Chitin/Collagen Scaffolds

A full schematic of the whole synthetic process is reported in Figure 1.

Initially, a 1 mg·mL^−1^ dispersion of chitin in acetic acid at pH 3 was obtained by setting 200 mg of chitin (cut into 4–6 mm^2^ pieces) in a beaker and adding 200 mL of solvent. The dispersion was then stirred with a blender for 10 min until a homogenous viscous dispersion was obtained.

In parallel, a 1 mg·mL^−1^ dispersion of collagen was obtained by setting 250 mg of collagen in a beaker with 50 mL of acetic acid at pH 3. The dispersion was stirred overnight and then centrifuged (2000× *g* for 5 min) to separate the soluble fraction from the insoluble one. The process was repeated two more times, stirring for 3 h each, and the soluble fractions were collected in a single batch. Then, the final pellet was dried and weighed to define the final concentration of the collagen in the dispersion. The collagen was then diluted to obtain the concentration desired.

The chitin and collagen dispersions were then mixed in the desired, homogenized with a vortex, frozen with liquid nitrogen, and freeze-dried to obtain a fibrous fluff. In this study, we refer to the dispersion, relative fluff, and scaffolds with different mass ratios as follows:

- C00: 100% collagen dispersion;
- C25: 25% chitin and 75% collagen;
- C50: 50% chitin and 50% collagen;
- C75: 75% chitin and 25% collagen;
- C100: 100% chitin dispersion.

The freeze-drying process was performed using a FreeZone 1 (Labconco Corp., Kansas City, MO, USA). The fluff was then redispersed in acetic acid, pH 3, to obtain a 10 mg·mL^−1^ dispersion. A desired volume of the dispersion was then cast in a multiwell, frozen with liquid nitrogen, and freeze-dried to obtain the final 3D scaffold.

The scaffold was then soaked in water for 10 min; then, the solvent was eliminated, and this washing step was repeated two more times. The scaffold was then frozen in liquid nitrogen, freeze-dried, and stored dry at 4 °C.

### 2.4. Infrared Spectroscopy

The attenuated total reflectance Fourier-transform infrared spectroscopy (ATR-FTIR) spectra were collected using a Nicolet IS10 spectrophotometer. The dry samples were analyzed by ATR with 2 cm^−1^ resolution and 100 scans using a germanium crystal. Omnic software 9.8.286 (Thermo Electron Corp., Woburn, MA, USA) was used for data processing.

### 2.5. Exposed Surface Measurement

The total surface area was determined using a BET (Brunauer, Emmett, and Teller) specific surface area analyzer Gemini VII 2390p Series (Micrometrics Instruments Corporation, Norcross, GA, USA) and evaluating the adsorption of N_2_ on the scaffold. The measurement was performed by applying an equilibration time of 10 s and an evacuation rate of 200 mmHg·min^−1^. The measure was carried out on at least four independent samples.

### 2.6. Water Absorption (WA) and Total Pore Volume (TPV) Percentage Measurements

The different scaffolds’ WAs were determined by weighing a dry matrix (stored overnight in a desiccator with dry CaCl_2_), soaking the matrix in water for 24 h, blotting it with a paper towel to remove surface water droplets, and weighing it again. The WA was determined as a ratio between the wet weight and the dry weight; the percentage was then calculated.

The total pore volume (TPV) was determined assuming the scaffold was a perfect cylinder. The diameter and height of the scaffold, used to determine the scaffold volume, were determined with a caliper (±0.05 mm) on a wet scaffold. It was assumed that all the water in the scaffold was contained in the scaffold pores and only a neglectable amount was present as structural water. The water mass present was derived from the wet weight calculated in the WA measurement minus the dry weight of the scaffold; the volume was calculated considering a water density of 1 g·mL^−1^. The total pore volume was calculated as a ratio between the water volume present and the scaffold volume; the percentage was then calculated.

Both the WA and TPV measurements were performed on four independent samples of about 20 mg of dry weight, except for sample C00, which was tested in duplicate. Each specimen’s weight was measured two times, and the average was used. Mass measurements were performed using a Sartorius CP225D (±0.01 mg).

### 2.7. Scanning Electron Microscopy (SEM)

SEM images were acquired with a LEO 1530 FEG, Zeiss, Oberkochen Germany using a tension of 5 kV. The dry samples were glued on carbon tape, stored overnight in a desiccator, and coated with 20 nm of gold prior to imaging them.

### 2.8. Mechanical Compression Tests

Compression tests were performed using a universal testing machine (Mod. 4465 with Series IX software, Instron, Norwood, MA, USA). The tests were performed with an actuator speed of 2 mm·min^−1^ (resulting in a strain rate of about 20%·min^−1^) at room temperature. The specimens tested were cylindrical with a 9–11 mm height, about a 14 mm diameter, and about a 20 mg dry weight. The samples were tested dry and wet (being previously soaked in water). For each class of sample, at least four independent samples were tested.

The compression curve allowed us to extrapolate the following parameters:

Young’s modulus: Young’s modulus was calculated by linear interpolation in the 2–25% deformation range, where the curve appears to have a linear trend.

Tenacity: Tenacity was calculated as the area of the compression profile in the 2–25% deformation range, where an elastic behavior was observed.

Densification: The densification point was determined as the last point after the curve derivative was maintained at 0.6 for at least six consecutive points. At this point, both deformation and stress were determined.

Data analyses were performed using MatLab. Statistical analyses were performed using an independent samples *t*-test with *p* = 0.05.

### 2.9. Cell Cultures

Human Osteosarcoma (MG63—passage #11) cells, human keratinocytes (HaCaT—passage #19), and mouse embryonic fibroblast (NIH-3T3/GFP—passage #27) cells were cultured under standard conditions in Dulbecco Modified Eagle’s Medium (DMEM), supplemented with 10 vol.% fetal bovine serum, 0.1 mM MEM Non-Essential Amino Acids (NEAA), 100 U·mL^−1^ penicillin, and 100 U·mL^−1^ streptomycin. The scaffolds were sterilized with UV light and presoaked in the culture medium prior to use. The cells were seeded on a scaffold in 24-well plates and 96-well plates at a density of 10^5^ cells·mL^−1^. Specifically, for MG63 cells, after cell adhesion, each sample was supplemented with 10 µg·mL^−1^ ascorbic acid and 5 mM β-glycerophosphate for osteoblast activation. MG63 cells were incubated for 2, 7, 14, and 21 days in a humidified incubator set at 37 °C in an atmosphere of 5% CO_2_. NIH-3T3 and HaCaT were incubated for 24, 48, and 72 h. Each experiment was repeated in triplicate, and five replicates were performed for each sample.

### 2.10. Cell Viability Test Using a Resazurin Reduction Assay

Cell viability was determined by the resazurin reduction assay, an oxidized form of a redox indicator that is blue in color and non-fluorescent. Briefly, the cells were seeded on scaffolds with a complete medium. After incubation times, resazurin reagent was added directly to the culture medium at 10% volume of the medium contained in each sample and incubated for 4 h at 37 °C with 5% CO_2_ [72]. Subsequently, aliquots from each sample were transferred to a 96 multiwell plate for fluorescence measurement at λ_exc_ 560 nm, em/λ_em_ 590 nm (Thermo Scientific Varioskan Flash Multimode Reader, Thermo Fisher Scientific, Waltham, MA, USA). We included a negative control of only the medium without cells to determine the background signal and a positive control of 100% reduced resazurin reagent without cells [73].

### 2.11. Fluorescence Microscopy on Osteoblasts

MG63 cells were fixed with 4 vol.% paraformaldehyde in DPBS and washed with DPBS. They were then permeabilized with 0.001% Triton-X 100 (Merck, Darmstadt, Germany). The cells were labeled with TRITC-conjugated phalloidin (FAK100, Merck Millipore, Burlington, MA, USA) for 1 h and then rinsed with DPBS. Actin staining was critical to map the local orientation of actin filaments within the cells. Nuclear counterstaining was performed by incubation with DAPI (FAK100, Merck Millipore) for 3 min, followed by rinses with DPBS [74]. NIH-3T3/GFP cells were observed directly by green fluorescence protein (GFP) after 72 h of cell incubation. Samples were examined using a Nikon Eclipse 80i microscope equipped for fluorescence analysis.

## 3. Results

### 3.1. Preparation of the Scaffolds

This work focused on the study of 3D scaffolds obtained by mixing and concentrating the dispersion through freeze-drying a chitin dispersion and a collagen dispersion.

The chemical and structural features of the chitin dispersion were verified by performing XRD and FTIR on the dried dispersion. The data showed signals coherent with the β-chitin polymorph; see Figure S1. Moreover, when observed using an optical microscope, the viscous chitin dispersion appeared free of micrometric particles, as shown in Figure S1, and no residue was collected when filtering on a 0.5 mm mesh tissue. For this reason, the yield of the dissolution was considered 100%. In a previous work by our group, Barbalinardo et al. (2021), a similar chitin dispersion was obtained using magnetic stirring instead of mechanical stirring [24]. This protocol modification shortened the time required for the dispersion preparation from 72 h to 10 min. In the same previous work, a study propaedeutic to this one explored the effect of the assembly of 2D chitin and collagen composites on cell adhesion but did not focus on an appropriate material characterization or a material format adequate for practical application, which is only addressed in this manuscript.

**Figure 2:**
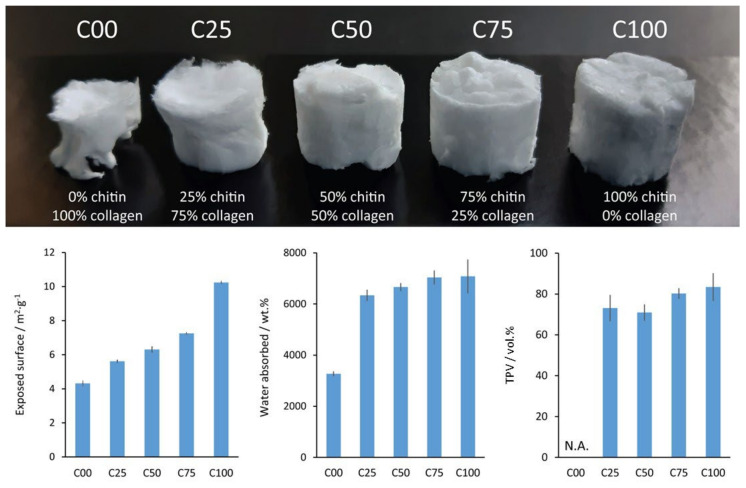
(**Top**) Camera picture of the scaffolds synthesized; each sample is about 1.4 cm in diameter. (**Bottom**) Histograms reporting the exposed surface measured using BET, the water absorbed, and the volume occupied by the pores in the scaffold. The total volume occupied by the pores in C00 was not calculated since this measure assumes a cylindrical geometry of the sample. N.A. = not applicable (due to geometric limitations).

Here, the 1 mg·mL^−1^ collagen dispersion and the 1 mg·mL^−1^ chitin dispersion were mixed using different volume (and, consequently, weight) ratios of the biopolymers, as reported in Figure 2. This allowed us to prepare final materials with different compositions and explore the material properties associated with them. Although collagen is generally soluble in this condition, due to its high concentration, it will form soluble nano-aggregated forms. For this reason, we refer to that as a dispersion and not as a solution [26].

Once the dispersion was obtained, the mixture was freeze-dried. The obtained foams were all too soft, unable to sustain any manipulation, and easily damaged when exposed to water. For this reason, an aliquot of a new solvent (acetic acid pH 3) was added to obtain a concentrated dispersion of 10 mg·mL^−1^, cast in a multiwell, and freeze-dried again. The obtained matrices showed proper mechanical and structural stability and were not soluble in water even after long exposure or manipulation (including the pure collagen matrix). It was not possible to directly blend the dispersions into a 10 mg·mL^−1^ concentration since the obtained dispersions would have been in-homogeneous (in terms of fiber dimension) and extremely viscous (thus, hard to mix into a desired ratio). After these steps, all the matrices showed a shape and dimension like the well in which the dispersion was cast.

The solvent used in the dispersion was recovered at the end of each freeze-drying step (Figure 1). During a freeze-drying cycle, about 92 vol.% of the solvent was re-collected with an acetic acid content (evaluated by pH measurements) of 53 mol.%. Then, the matrices were washed by soaking them multiple times in water. During this step, C00 showed significant shrinkage associated with rehydration. This deformation was observed more in the samples with a higher collagen content. Despite that, all the samples containing chitin appeared to preserve their shapes after being fully rehydrated, while C00 maintained a distorted morphology. The samples were then dehydrated by freeze-drying. A picture of these different matrices, prepared in a 24-multiwell plate, is shown in the top panel of Figure 2.

The ATR-FTIR spectra (Figure S2 and Table S1) of C100 and C00 showed the typical absorption bands of chitin and collagen, respectively, as reported in the literature [24]. Chitin showed intense absorption bands for amide I (1648 cm^−1^) and II (1560 cm^−1^), CH bending (1377 cm^−1^), four signals related to glycosidic ring stretching (1151, 1113,1070, and 1034 cm^−1^), N−H stretching (3293 cm^−1^), and O−H stretching (3420 cm^−1^) [29]. The major absorption bands associated with collagen were amide I (1653 cm^−1^), II (1544 cm^−1^), III (1239 cm^−1^), A (3323 cm^−1^), and B (3079 cm^−1^) [26]. When moving from C100 to C00, an increase in the relative intensity of the collagen absorption bands was observed, along with a decrease in chitin ones. The absorption band shifts observed could be associated with the overlapping chitin and collagen bands due to the varying sample compositions or to a band shift due to non-specific interactions between the two biopolymers. These observations are coherent with those of a previous study by our team, where the interaction between these two biopolymers in similar preparation conditions was examined [24]. The study showed the interaction between these two polymers is likely due to non-specific hydrogen bond, electrostatic, or apolar region interactions. Despite that, the study showed these two components combine into a nano-homogeneous material. Considering that the same band pattern is observed in these samples, we can assume a nano-homogeneous composite was obtained here, too.

### 3.2. Exposed Surface, Water Absorption, and Total Pore Volume

The dry scaffolds were then characterized using BET to determine their exposed surfaces. The results showed an incremental trend when increasing the amount of chitin present. A linear trend was observed when moving from sample C00 (4.3 m^2^·g^−1^) to sample C75 (6.3 m^2^·g^−1^). The following equation was identified with an R^2^ = 0.985.

[Exposed surface] = 0.038 × [Chitin percentage] + 4.443.

C100 diverged from this trend, showing a higher exposed surface (10.2 m^2^·g^−1^), about two times that of C00 and C25. The BET analysis data are reported in Figure 2 and Table S2.

The WA, on the other hand, was observed to have a much different behavior. Samples C75 and C100 showed no difference, while an incremental trend from about 6300% to 7000% was observed when moving from C25 to C75. Despite that, among them, the only significantly different samples were C25 and C75 (*t*-test, *p* = 0.05, ν ≥ 6). C00 was significantly different from all the other samples (*t*-test, *p* = 0.05, ν ≥ 6) and showed almost half the WA of C25, being about 3200%. The WA data are reported in Figure 2 and Table S2. FT-IR spectra were also collected after the swelling measurements, showing no significant changes in the absorption bands. This suggests a compositional stability of the scaffold in water.

In Figure 2 and Table S2, we refer to TPV as the volume of the scaffold that is likely occupied by water or air when dried. Since the measure of this parameter implies assuming a perfectly cylindrical shape, the deformed morphology of C00 did not allow us to determine these data. The other scaffolds showed a value of about 72% for samples C25 and C50 and about 82% for C75 and C100.

### 3.3. Morphology and Mechanical Tests

**Figure 3:**
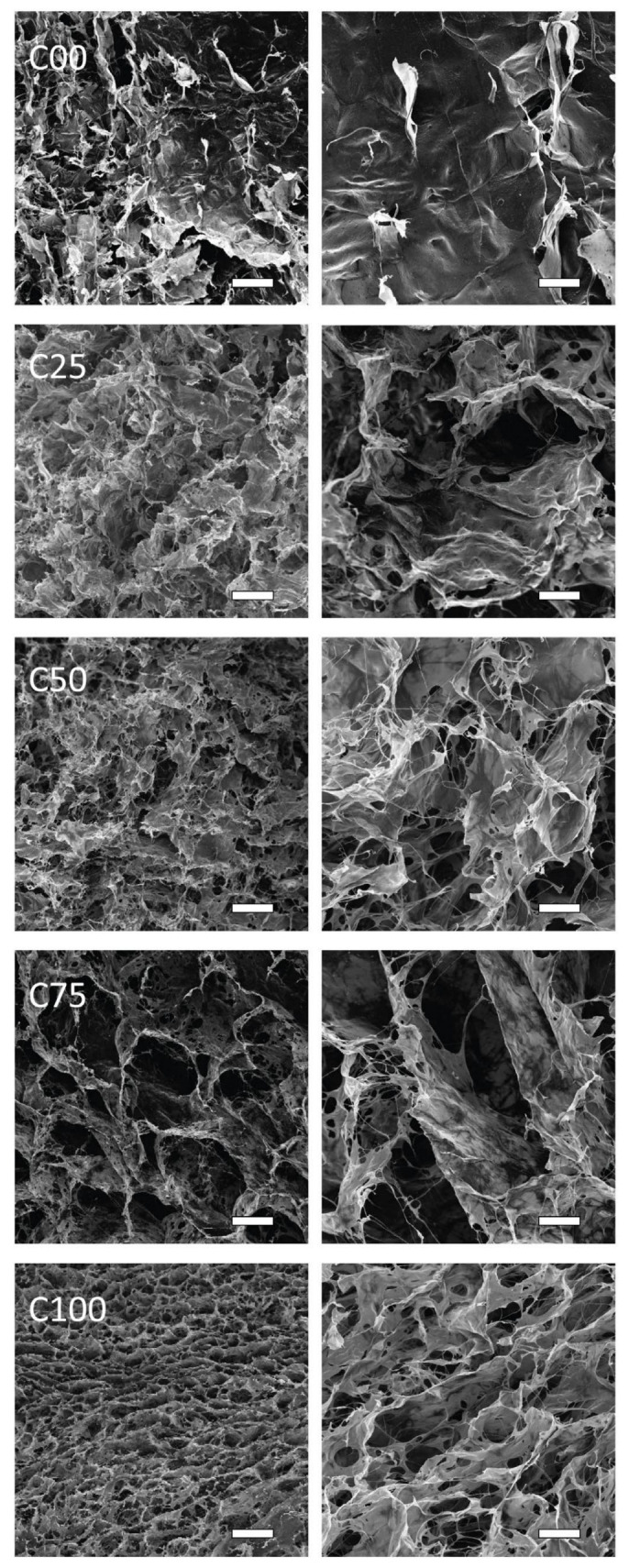
SEM images of a section of the scaffolds synthesized. On the left, a lower magnification (scale bar 100 μm); on the right, a higher magnification (scale bar 30 μm).

The SEM investigation of the different matrices showed their porous structures. As can be observed in Figure 3, the pore cavities became bigger when moving from C00 to C75 and then decreased again in C100. Observing the matrices using a higher magnification, a more fibrous appearance was observable when increasing the relative amount of chitin. This led to porous pore walls and a less defined border of the cavity. The increase in the amount of collagen appeared to contribute to a more compact and sheet-like organization with neglectable micro-porosity. Due to the irregular shape of the pores observed by SEM, a pore dimension could not be estimated.

**Figure 4:**
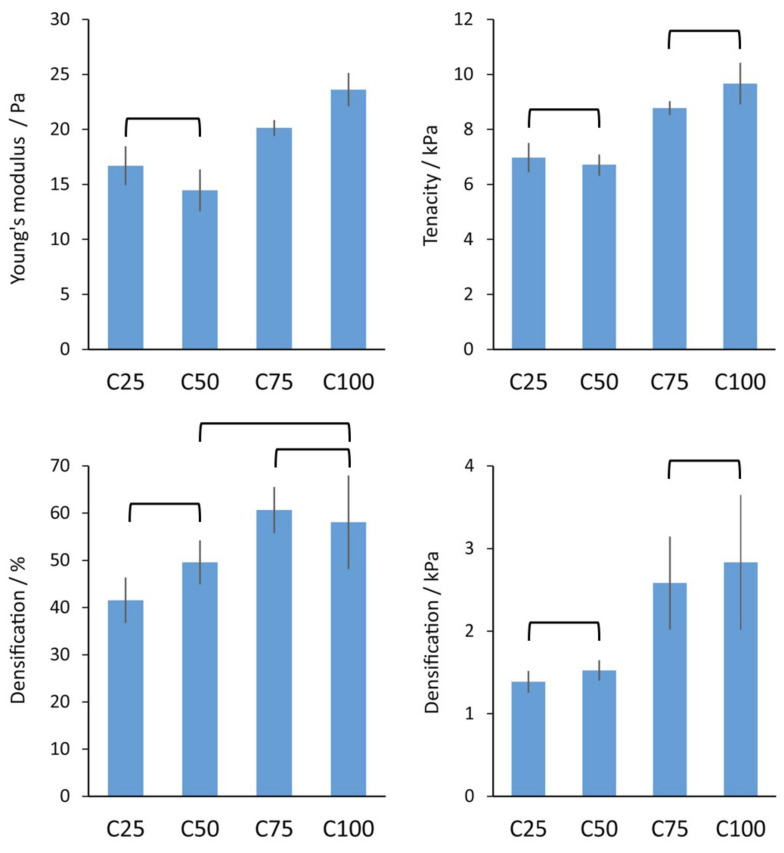
Comparison of the mechanical parameters obtained from wet compression tests of the scaffolds. Because of the geometry of the scaffold, it was not possible to obtain reliable data on the C00 scaffold. Data are presented as mean ± standard deviation (SD). The statistical analyses were performed using a *t*-test (*p* = 0.05, ν ≥ 6). Each graph connection denotes no significant difference in the couple.

Compression tests were performed on all the matrices except C00. This is because the data analyses were performed assuming a cylindrical geometry of the sample, and it would not have been possible to extract any reliable data on C00. When tested, a strong positive contribution of chitin was observed. When moving from C25 to C100, a general increase in Young’s modulus (YM, from about 17 to 24 Pa), tenacity (from 7 to 10 kPa), and densification (in terms of deformation, from about 40% to 60%, and stress, from about 1.4 to 2.8 kPa) were observed. For each parameter, the sample group was tested using a *t*-test. The tests showed C25 and C50 to be significantly equal in all the parameters measured, while only a difference in Young’s modulus was observed between C75 and C100. The results of the compression tests are reported in Figure 4, Table S3, and Figure S3 (raw curves).

### 3.4. Cell Viability of Keratinocytes, Fibroblasts, and Osteoblasts

**Figure 5:**
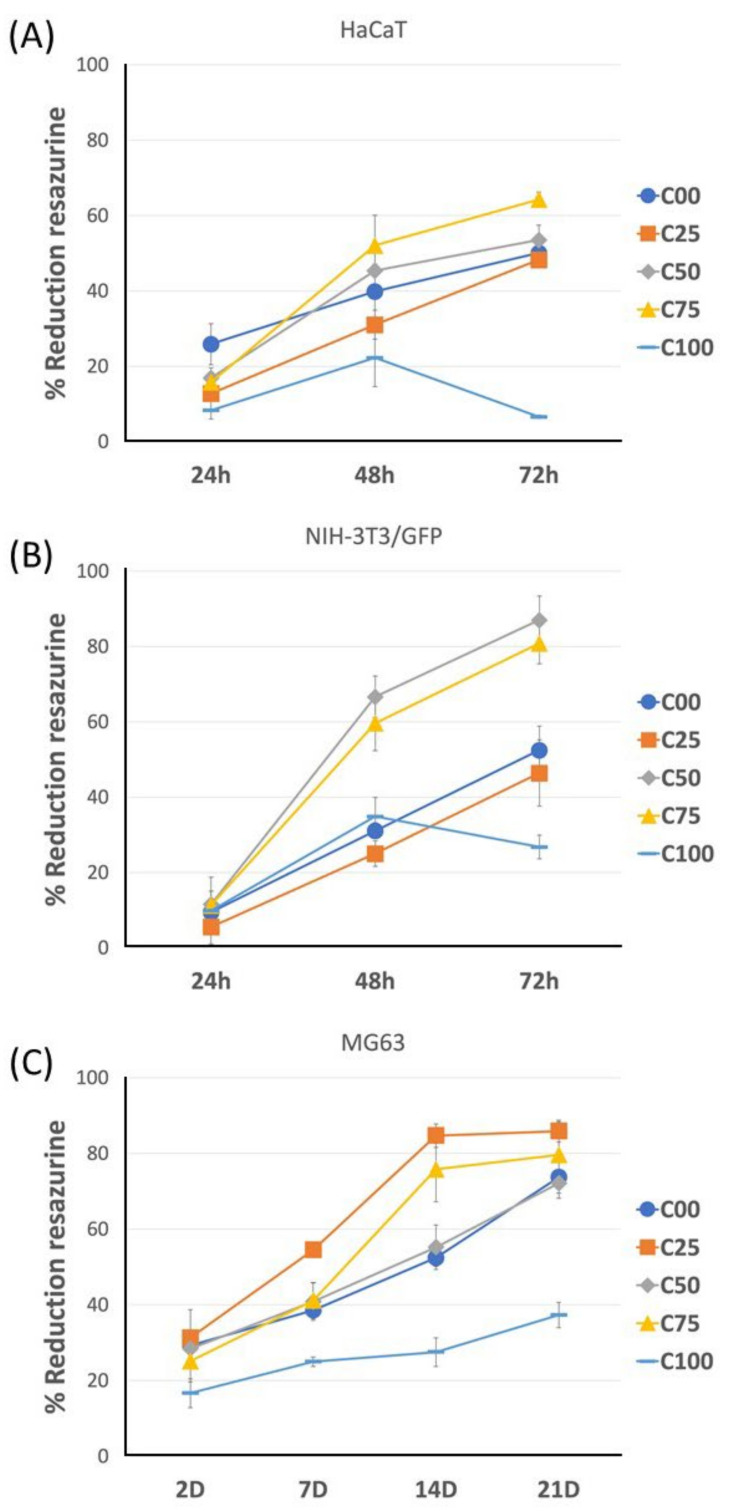
Cell viability calculated by resazurin reduction of HaCaT (**A**) and NIH-3T3 (**B**) cells after 24 h, 48 h, and 72 h and MG63 (**C**) after 2, 7, 14, and 21 days on scaffolds. Data are presented as mean ± standard deviation (SD).

The biocompatibility of the scaffolds prepared was tested by evaluating the viability of different cell types cultured in vitro on the scaffold. For this study, three different cell typologies were examined: keratinocytes, fibroblasts, and osteoblasts. The viability data, reported as a reduction of resazurin, are reported in Figure 5.

Keratinocytes (Figure 5A) showed an incremental trend in viability when collagen was present, while a drop in viability was observed on the third day for sample C100. At 24 h, the highest viability was observed for sample C00, followed by C50, C75, C25, and C100. Successively, an inversion was observed at 48 h and maintained at 72 h. At the end of the experiment, the highest viability was observed for sample C75 (64%), followed by C50 (54%), C00 (50%), C25 (48%), and C100 (7%).

Fibroblasts (Figure 5B) showed a completely different behavior at 24 h, generally with the samples with a higher chitin content showing the highest viability, decreasing from C50, C75, C100, C00, to C25. This same sequence was maintained up to 72 h, except for sample C100, which showed a drop in viability at 72 h. At the end of the experiments, the highest viability was recorded for C50 (87%), followed by C75 (81%), C00 (52%), C25 (46%), and, finally, C100 (27%). Figure S4 shows the green fluorescence protein (GFP) fluorescence of scaffold-attached fibroblasts after 72 h. In agreement with the viability data for samples C50 and C75, we observed a higher number of attached cells.

**Figure 6:**
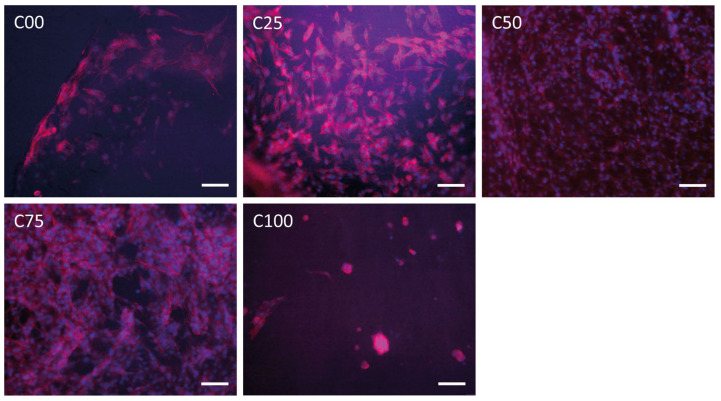
Fluorescence micrographs of osteoblast labeled specifically for actin (red) and the nucleus (blue) after 14 days on scaffolds (scale bar: 100 μm).

Osteoblasts (Figure 5C) are not a type of cell of interest from a biological point of view for this study. These cells were tested to evaluate cell proliferation over a long time due to their slow proliferation, thus identifying the eventual long-term toxicity of the material. After 2 days, the highest viability was observed for C25 (31%), followed by C00 and C50 (28% and 29%), C75 (25%), and C100 (17%). Between 2 and 7 days, C75 showed a high increment in viability. From 7 to 21 days, a constant increment in viability was observed for all the samples, including C100. At the end of the experiment, the highest viability was observed for C25 (86%), followed by C75 (80%), C00 and C50 (74% and 72%), and C100 (37%). In agreement with the viability results, the double fluorescence labeling (Figure 6) of actin (red) and nucleus (blue) showed high osteoblast adhesion and proliferation after 14 days in the C25, C50, and C75 samples. Instead, in the C100 sample, the presence of chitin alone did not favor the adhesion of cells that grow in clusters, preferring to proliferate one above the other.

## 4. Discussion

### 4.1. Scaffold Preparation

In this work, a set of green composite materials is proposed for medical applications. These materials are fully synthesized in a mild water-based solvent. This is especially new for chitin since it is usually dispersed using concentrated acid/base [75,76] or very polar organic solvents [46,51,62].

It is worth mentioning that this work focuses on actual chitin (with a degree of acetylation of about 89%) and not on chitosan (a highly deacetylated form of chitin, with a degree of acetylation below 30%). These two biopolymers are often confused with each other [49,54,63] but are deeply different. Compared to chitin, chitosan dissolves in mildly acid environments due to its high number of free amino groups (pKa of about 6.3 [77,78,79]), which represents a strong limitation in its application. In fact, chitosan is known to lose most of its structural properties and mechanical resistance once exposed to a pH slightly below 7 [80]. This makes it an intriguing material for drug delivery on inflamed tissues but limits its application for grafting or wound dressing since it would dissolve or lose structural integrity, on an inflamed tissue (which is slightly acid). For this reason, wound dressings based on chitosan are also generally reported as cross-linked, which, as a consequence, limits their biodegradability and includes highly reactive and potentially toxic components in the scaffold [60,81]. On the other hand, chitin is inert to acid pH, and its dissolution in our solvent was possible due to the high mechanical stress imposed on the solution and the open morphological structure of the initial squid pen [24,39]. Once deposited and washed, the scaffold was completely inert in the same dissolution conditions.

The β-chitin nanofibril dispersion was produced using a process documented for the first time by Fan et al. (2008) [82] and then further perfectioned in our research group [24,39]. This allows for dispersing β-chitin in a pH 3 solution of acetic acid (56 mM or 3.2 vol.%), a condition that is also able to solubilize collagen. Compared to previous works [24,39], here, we used mechanical stirring to quickly obtain the chitin dispersion, compared to slower magnetic stirring, and with lower energetic requirements than sonication. XRD, FTIR, and optical microscopy data showed how a submicron fiber dispersion was obtained and how the β-chitin polymorph was preserved, meaning that no complete solubilization of the polymer occurred [83]. In addition, the choice of this protocol is especially important since acetic acid is a biological molecule that is fully bio-compatible and eco-compatible in small amounts. Moreover, the concentration used is technically addressed as an “irritant”, even though it could be safely consumed as it is less concentrated than commercial vinegar (usually about 4–5 vol%), so its handling can be considered safe.

Using this methodology, it was possible to disperse chitin and collagen, obtaining a nanometric-scale homogeneous material, as we reported in Barbalinardo et al. (2021) [24]. Different 3D scaffolds were then obtained by freeze-drying the dispersions with different relative amounts of the two components. The scaffold composition was checked using FTIR, observing analogous peaks, as in our previous study, thus suggesting that a nano-homogeneous material was obtained.

All scaffolds appeared stable, showing no visible alteration in water or in the cell-growing medium for more than 20 days. Despite that, the one fully made of collagen, C00, strongly deformed once exposed to water, suggesting the importance of chitin in the structural integrity of the material.

An interesting advantage given by this synthetic approach is the recyclability of the solvent; see Figure 1. No stochiometric chemical is consumed to produce the final material, and only acetic acid and water are used to promote the biopolymer dispersion. Since both acetic acid and water are highly volatile in freeze-drying conditions, it is possible to recover most of the solvent (92 vol.%) after each dehydration step. This solvent could be used again to produce a new dispersion (an alteration of pH of about 0.1 was observed), leading to almost no solvent waste except the washing waters (which are slightly acidic). The minor loss in solvent volume observed may be due to vapors ending in the vacuum pump; this loss could be minimized by fine-tuning the dehydration conditions and experimental setup. On the other hand, a decrease in the acetic acid concentration was observed. This may be due to a lower volatility of the acid compared to water and a higher retention of the acid in the matrix. The missing acid is about 0.1 vol.% of the total volume of the solvent; thus, it is neglectable in terms of solvent volume recovery. The missing acid is likely retained in the structure during dehydration and is then washed away during the washing steps. The final content of acid in the matrix was not evaluated. It is worth mentioning that no color change was observed in the cell medium during the cell culture tests, suggesting no relevant acid traces were present in/released from the final scaffold.

### 4.2. Material Characterization

A higher exposed surface, WA, TPV, and wet mechanical resistance were observed when increasing the relative amount of chitin in the scaffold.

Overall, a WA of 6000–7000 wt.% and a TPV of 70–80% were observed in the scaffold with a 25–100 wt.% content of chitin. In terms of porosity, this protocol seems to provide a material with values analogous to that reported by Lee et al. (2004) [50], i.e., 63–78%, who used DMAc and LiCl as solvents for scaffold production. A similar value was also reported by Sudheesh Kumar et al. (2011) [58], i.e., about 80% for pure chitin, who instead prepared a chitin solution using CaCl_2_ and methanol as the solvent. The authors also reported a swelling of about 2000–2400%, which is about one-third of the one reported for our C100. Despite what was claimed by the authors, the X-ray diffraction peak showed a signal at about 9–10°, which is coherent with the α-chitin polymorph. The difference in swelling observed in our scaffold may be attributed to the presence of β-chitin in the scaffold instead of α-chitin. It is interesting to observe that even in the sample with the lowest amount of chitin, C25, the presence of chitin was able to almost double the WA of the scaffold compared to pure collagen, C00. This difference is probably due to the massive shrinkage that the C00 scaffold goes through when exposed to water, which drastically diminishes its volume, and also to its 3D organization.

The SEM observation of the scaffolds showed, in fact, coherent results with the other parameters studied. A general increase in pore cavities was observed when increasing the amount of chitin. Such an increase in porosity is coherent with the results of WA and TPV, especially when moving from C00, showing a more compact material, to C25, exhibiting visible porosity. After that, the SEM images showed a mild increase in porosity when moving from C25 to C100, as expected when looking at the values of WA and TPV. On the other hand, a relevant change in the pore wall morphology was observed when increasing the chitin content. This change led to numerous thin fibrous walls in C100 other than the sheet-like walls in the collagenous samples. Such a modification would explain why the exposed surface was so affected by the chitin content, while only minor changes were observed in WA and TPV. Although both chitin and collagen are known for their fibrous behavior, chitin is still organized into crystalline fibrils with a much longer aspect ratio compared to collagen molecules. This easily explains the fibrous morphology associated with this specific component.

Combining the morphological analyses with the compression test results, it is noticeable how chitin increases the stiffness, energy absorption, and compressibility of the scaffolds (as shown in the trends in YM, tenacity, and densification) despite thinner and more micro-porous pore walls being present. This observation is also coherent with the crystalline nature of chitin and the fact that collagen generally exhibits high mechanical properties once self-assembled into microfibers.

The results suggest that similar porosity, WA, and compression resistance could be achieved using materials with 25–50% β-chitin and 75–100% β-chitin, showing a step trend in the composite properties.

All the chitin-containing scaffolds exhibited high porosity, which is crucial for wound dressings since it facilitates the exchange of gasses through the material. This positively combines with the high WA and mechanical resistance in wet conditions, which is crucial when treating exudating wounds such as burn injuries, where the dressing needs to be able to handle high fluid production from the wound. In regenerative medicine, on the other hand, the high porosity would also benefit nutrient diffusion while ensuring a tight interaction with the surrounding tissues. Moreover, the highly exposed surface would make these scaffolds intriguing candidates as drug delivery systems.

### 4.3. In Vitro Cell Tests

In this study, three different cell typologies were cultured on the scaffolds: fibroblasts, keratinocytes, and osteoblasts. In these tests, the similarity between samples C75 and C100 observed in the material’s properties is completely lost, showing how important collagen is for appropriate interaction with cells.

Although C100 showed the worst viabilities for all the three cell types tested, no toxic effect was observed using this sample, and cells were also able to proliferate on this scaffold. This low cell viability is a consequence of low cell adhesion, which is due to the low surface wettability of chitin compared to other biopolymers (showing a contact angle of about 80° [71]). Cells in the presence of materials with a surface tension greater than 60° begin to have difficulty adhering. In fact, as we can observe in Figure 6, in sample C100, the cells do not adhere properly and assume a rounded morphology diagnostic of low adhesion. The cells that adhere continue to proliferate, which are, in this case, osteoblasts that prefer to build a 3D structure on the same cell matrix.

Contrary to C100, all the composites outperformed the two pure materials, showing a synergic effect of the components on their cell interactions.

Fibroblasts showed a higher viability on C50 and C75, observable in the first 24 h. On the other hand, the preference for these scaffolds was observed in keratinocytes between 24 and 48 h. While the first showed a light preference for C50, the second showed instead a preference for C75. These two matrices appeared to give the best results, even better than pure collagen, at 72 h on both cell lines. This is especially important in view of a wound dressing application. In fact, these cells represent the skin’s main components, as much as the ones mostly involved in skin healing [84,85]. Considering that the data suggest a concentration of 50–75 wt.% of chitin may be the best one for this application.

The scaffolds were also tested using osteoblasts, which are not the biological target of these scaffolds. These cells are very slow in their development and thus allowed us to test the scaffolds for a very long time without reaching cell overpopulation. None of the scaffolds gave any sign of cytotoxicity, and the cells were able to develop in a uniform layer. The only exception was observed in C100, where the cells, having poor adhesion with chitin, grew and proliferated in clusters. This test is very important for the validation of the protocol used for the preparation of the scaffolds since it demonstrates that it does not leave any harmful contaminants in them. As for the previous tests, the composites were able to surpass or equal pure collagen.

In general, diverse preferences in the relative amount of the two components were observed to be more effective towards different types of cells, suggesting a customized composition should be used to target different tissues.

## 5. Conclusions

A novel method for the green synthesis of biodegradable and bio-compatible materials obtainable from renewable resources (also extractable from alimentary and textile wastes) is proposed. Compared to previous analogous works, both chitin and collagen are dispersed in just 10 min using a mild acetic acid aqueous solution (with an acidity below that of commercial vinegar). In addition, the process used exhibits a high solvent recovery (>90 vol.%), lowering the environmental impact of this production process.

The composite scaffolds prepared are composed of different ratios of collagen I and β-chitin, a polymorph known for its high wettability that is rarely retained after chitin solubilization. The data collected on these 3D composites show, for the first time, the influence of composition on the material properties and their biological effects. A high positive influence of the chitin content (in the 0–100 wt.% range) on the exposed surface of the material (up to 7 m^2^·g^−1^) and its wet mechanical properties is observed. On the other hand, limited improvements are observed in water absorption (up to 7000 wt.%) and total pore volume (over 80 vol.%) when increasing the chitin content above 25 wt.%. Despite that, all the parameters discussed increase with the amount of chitin in the scaffold, highlighting a step trend with similar properties for samples with 25–50 wt.% chitin and 75–100 wt.% chitin. The scaffolds were then tested as substrates for fibroblast, keratinocyte, and osteoblast growth. All the composites showed higher or equal cell viability compared to pure collagen or chitin, suggesting a synergic effect of the two components. Mass ratios of 50–75 wt.% of chitin gave the best results in terms of viability and cell adhesion. No material showed signs of cytotoxicity even after 21 days.

We believe this novel protocol may find a wide application in substituting preparations with a higher ecological impact in the medical sectors. Specifically, the novel materials presented have suitable properties to be highly performing green alternatives for wound dressings (a medical disposable waste), especially when prepared with a 50–75 wt.% chitin content.

## Data Availability

The submitted manuscript materials, data, and associated protocols will be made available to readers by the corresponding author.

## Supplementary Materials

The following supporting information can be downloaded at https://www.mdpi.com/article/10.3390/polym17020140/s1. Figure S1: Characterization of the β-chitin fibril dispersion. Figure S2: ATR-FTIR analysis of the porous scaffolds obtained. Table S1: FTIR absorption bands of the scaffolds studied. Table S2: Numerical values of the exposed surface, water absorbed, and the total pore volume. Table S3: Compression tests on the wet porous scaffolds. Table S4: Compression tests on the dry porous scaffolds. Figure S3: Compression profiles of the different specimens tested. Figure S4: Fluorescence micrographs of fibroblast after 72 h.

## References

[1] Ghersin Z.J., Flaherty M.R., Yager P., Cummings B.M. (2020). Going Green: Decreasing Medical Waste in a Paediatric Intensive Care Unit in the United States. New Bioeth..

[2] Conrardy J., Hillanbrand M., Myers S., Nussbaum G.F. (2010). Reducing Medical Waste. AORN J..

[3] Guest J.F., Ayoub N., McIlwraith T., Uchegbu I., Gerrish A., Weidlich D., Vowden K., Vowden P. (2017). Health Economic Burden That Different Wound Types Impose on the UK’s National Health Service. Int. Wound J..

[4] Guest J.F., Fuller G.W., Vowden P. (2018). Venous Leg Ulcer Management in Clinical Practice in the UK: Costs and Outcomes. Int. Wound J..

[5] Guest J.F., Fuller G.W., Vowden P. (2018). Diabetic Foot Ulcer Management in Clinical Practice in the UK: Costs and Outcomes. Int. Wound J..

[6] Guest J.F., Fuller G.W., Vowden P., Vowden K.R. (2018). Cohort Study Evaluating Pressure Ulcer Management in Clinical Practice in the UK Following Initial Presentation in the Community: Costs and Outcomes. BMJ Open.

[7] Guest J.F., Fuller G.W., Vowden P. (2018). Costs and Outcomes in Evaluating Management of Unhealed Surgical Wounds in the Community in Clinical Practice in the UK: A Cohort Study. BMJ Open.

[8] Agarwal A., McAnulty J.F., Schurr M.J., Murphy C.J., Abbott N.L. (2011). Polymeric Materials for Chronic Wound and Burn Dressings. Advanced Wound Repair Therapies.

[9] Kirwan H., Pignataro R. (2016). The Skin and Wound Healing. Pathology and Intervention in Musculoskeletal Rehabilitation.

[10] Nudelman F., Pieterse K., George A., Bomans P.H.H., Friedrich H., Brylka L.J., Hilbers P.A.J., De With G., Sommerdijk N.A.J.M. (2010). The Role of Collagen in Bone Apatite Formation in the Presence of Hydroxyapatite Nucleation Inhibitors. Nat. Mater..

[11] Alam P., Amini S., Tadayon M., Miserez A., Chinsamy A. (2016). Properties and Architecture of the Sperm Whale Skull Amphitheatre. Zoology.

[12] Seidel R., Jayasankar A.K., Dean M.N. (2020). The Multiscale Architecture of Tessellated Cartilage and Its Relation to Function. J. Fish Biol..

[13] Wenger M.P.E., Bozec L., Horton M.A., Mesquidaz P. (2007). Mechanical Properties of Collagen Fibrils. Biophys. J..

[14] Gentleman E., Lay A.N., Dickerson D.A., Nauman E.A., Livesay G.A., Dee K.C. (2003). Mechanical Characterization of Collagen Fibers and Scaffolds for Tissue Engineering. Biomaterials.

[15] Montroni D., Piccinetti C., Fermani S., Calvaresi M., Harrington M.J., Falini G. (2017). Exploitation of Mussel Byssus Mariculture Waste as a Water Remediation Material. RSC Adv..

[16] Nudelman F., Lausch A.J., Sommerdijk N.A.J.M., Sone E.D. (2013). In Vitro Models of Collagen Biomineralization. J. Struct. Biol..

[17] Montroni D., Valle F., Rapino S., Fermani S., Calvaresi M., Harrington M.J., Falini G. (2018). Functional Biocompatible Matrices from Mussel Byssus Waste. ACS Biomater. Sci. Eng..

[18] Montroni D., Giusti G., Simoni A., Cau G., Ciavatta C., Marzadori C., Falini G. (2020). Metal Ion Removal Using Waste Byssus from Aquaculture. Sci. Rep..

[19] Davison-Kotler E., Marshall W.S., García-Gareta E. (2019). Sources of Collagen for Biomaterials in Skin Wound Healing. Bioengineering.

[20] Zhang G., Young B.B., Ezura Y., Favata M., Soslowsky L.J., Chakravarti S., Birk D.E. (2005). Development of Tendon Structure and Function: Regulation of Collagen Fibrillogenesis. J. Musculoskelet. Neuronal Interact..

[21] Thorpe C.T., Screen H.R.C. (2016). Tendon Structure and Composition. Metabolic Influences on Risk for Tendon Disorders.

[22] Sell S.A., McClure M.J., Garg K., Wolfe P.S., Bowlin G.L. (2009). Electrospinning of Collagen/Biopolymers for Regenerative Medicine and Cardiovascular Tissue Engineering. Adv. Drug Deliv. Rev..

[23] Lin K., Zhang D., Macedo M.H., Cui W., Sarmento B., Shen G. (2019). Advanced Collagen-Based Biomaterials for Regenerative Biomedicine. Adv. Funct. Mater..

[24] Barbalinardo M., Biagetti M., Valle F., Cavallini M., Falini G., Montroni D. (2021). Green Biocompatible Method for the Synthesis of Collagen/Chitin Composites to Study Their Composition and Assembly Influence on Fibroblasts Growth. Biomacromolecules.

[25] Afifah A., Suparno O., Haditjaroko L., Tarman K. (2019). Utilisation of Fish Skin Waste as a Collagen Wound Dressing on Burn Injuries: A Mini Review. IOP Conf. Ser. Earth Environ. Sci..

[26] Tian Z., Wang Y., Wang H., Zhang K. (2020). Regeneration of Native Collagen from Hazardous Waste: Chrome-Tanned Leather Shavings by Acid Method. Environ. Sci. Pollut. Res..

[27] Matinong A.M.E., Chisti Y., Pickering K.L., Haverkamp R.G. (2022). Collagen Extraction from Animal Skin. Biology.

[28] Jafari H., Lista A., Siekapen M.M., Ghaffari-Bohlouli P., Nie L., Alimoradi H., Shavandi A. (2020). Fish Collagen: Extraction, Characterization, and Applications for Biomaterials Engineering. Polymers.

[29] Montroni D., Fermani S., Morellato K., Torri G., Naggi A., Cristofolini L., Falini G. (2019). β-Chitin Samples with Similar Microfibril Arrangement Change Mechanical Properties Varying the Degree of Acetylation. Carbohydr. Polym..

[30] Ogawa Y., Hori R., Kim U.J., Wada M. (2011). Elastic Modulus in the Crystalline Region and the Thermal Expansion Coefficients of α-Chitin Determined Using Synchrotron Radiated X-Ray Diffraction. Carbohydr. Polym..

[31] Vincent J.F.V., Wegst U.G.K. (2004). Design and Mechanical Properties of Insect Cuticle. Arthropod Struct. Dev..

[32] Montroni D., Palanca M., Morellato K., Fermani S., Cristofolini L., Falini G. (2021). Hierarchical Chitinous Matrices Byssus-Inspired with Mechanical Properties Tunable by Fe (III) and Oxidation. Carbohydr. Polym..

[33] Montroni D., Sparla F., Fermani S., Falini G. (2021). Influence of Proteins on Mechanical Properties of a Natural Chitin-Protein Composite. Acta Biomater..

[34] Montroni D., Zhang X., Leonard J., Kaya M., Amemiya C., Falini G., Rolandi M. (2019). Structural Characterization of the Buccal Mass of *Ariolimax californicus* (Gastropoda; Stylommatophora). PLoS ONE.

[35] Montroni D., Leonard J., Rolandi M., Falini G. (2021). Morphology and Organization of the Internal Shell of *Ariolimax californicus* (Gastropoda; Stylommatophora), an Asymmetric Two-Face Biomineralized Matrix. J. Struct. Biol..

[36] Huang W., Montroni D., Wang T., Murata S., Arakaki A., Nemoto M., Kisailus D. (2022). Nanoarchitected Tough Biological Composites from Assembled Chitinous Scaffolds. Acc. Chem. Res..

[37] Lee J.E., Connolloy J., Yang W., Freychet G., Wang T., Herrera S.A., Murata S., Dasika P.S., Montroni D., Pohl A. (2023). Fibrous Anisotropy and Mineral Gradients Within the Radula Stylus of Chiton: Controlled Stiffness and Damage Tolerance in a Flexible Biological Composite. J. Compos. Mater..

[38] Montroni D., Sarmiento E., Zhao R., Dasika P.S., Connolly J.M., Wuhrer R., Zhang Y., Zhernenkov M., Wang T., Ramirez-Santana B.P. (2024). The Multiphasic Teeth of Chiton Articulatus, an Abrasion-Resistant and Self-Sharpening Tool for Hard Algae Collection. Adv. Funct. Mater..

[39] Montroni D., Marzec B., Valle F., Nudelman F., Falini G. (2019). β-Chitin Nanofibril Self-Assembly in Aqueous Environments. Biomacromolecules.

[40] Montroni D., Di Giosia M., Calvaresi M., Falini G. (2022). Supramolecular Binding with Lectins: A New Route for Non-Covalent Functionalization of Polysaccharide Matrices. Molecules.

[41] Wan A.C.A., Tai B.C.U. (2013). Chitin—A Promising Biomaterial for Tissue Engineering and Stem Cell Technologies. Biotechnol. Adv..

[42] Abe M., Takahashi M., Tokura S., Tamura H., Nagano A. (2004). Cartilage—Scaffold Composites Produced by Bioresorbable b -Chitin Sponge with Cultured Rabbit Chondrocytes. Tissue Eng..

[43] Ge Z., Baguenard S., Yong L., Wee A., Khor E. (2004). Hydroxyapatite—Chitin Materials as Potential Tissue Engineered Bone Substitutes. Biomaterials.

[44] Magnabosco G., Ianiro A., Stefani D., Soldà A., Rapino S., Falini G., Calvaresi M. (2020). Doxorubicin-Loaded Squid Pen Plaster: A Natural Drug Delivery System for Cancer Cells. ACS Appl. Bio Mater..

[45] Jayakumar R., Nair A., Rejinold N.S., Maya S., Nair S.V. (2012). Doxorubicin-Loaded PH-Responsive Chitin Nanogels for Drug Delivery to Cancer Cells. Carbohydr. Polym..

[46] Montroni D., Kobayashi T., Hao T., Lublin D., Yoshino T., Kisailus D. (2022). Direct Ink Write Printing of Chitin-Based Gel Fibers with Customizable Fibril Alignment, Porosity, and Mechanical Properties for Biomedical Applications. J. Funct. Biomater..

[47] Okamoto Y., Watanabea M., Miyatakea K., Morimoto M., Shigemasa Y., Minami S. (2002). Effects of Chitin/Chitosan and Their Oligomers/Monomers on Migrations of Fibroblasts and Vascular Endothelium. Biomaterials.

[48] Mori T., Okumura M., Matsuura M., Ueno K., Tokura S., Okamoto Y., Minami S., Fujinaga T. (1997). Effects of Chitin and Its Derivatives on the Proliferation and Cytokine Production of Fibroblasts in Vitro. Biomaterials.

[49] Li X., Feng Q., Liu X., Dong W., Cui F. (2006). Collagen-Based Implants Reinforced by Chitin Fibres in a Goat Shank Bone Defect Model. Biomaterials.

[50] Lee S.B., Kim Y.H., Chong M.S., Lee Y.M. (2004). Preparation and Characteristics of Hybrid Scaffolds Composed of β-Chitin and Collagen. Biomaterials.

[51] Jin J., Hassanzadeh P., Perotto G., Sun W., Brenckle M.A., Kaplan D., Omenetto F.G., Rolandi M. (2013). A Biomimetic Composite from Solution Self-Assembly of Chitin Nanofibers in a Silk Fibroin Matrix. Adv. Mater..

[52] Arun Kumar R., Sivashanmugam A., Deepthi S., Iseki S., Chennazhi K.P., Nair S.V., Jayakumar R. (2015). Injectable Chitin-Poly(ε-Caprolactone)/Nanohydroxyapatite Composite Microgels Prepared by Simple Regeneration Technique for Bone Tissue Engineering. ACS Appl. Mater. Interfaces.

[53] Peter M., Sudheesh Kumar P.T., Binulal N.S., Nair S.V., Tamura H., Jayakumar R. (2009). Development of Novel α-Chitin/Nanobioactive Glass Ceramic Composite Scaffolds for Tissue Engineering Applications. Carbohydr. Polym..

[54] Li X., Feng Q., Wang W., Cui F. (2006). Chemical Characteristics and Cytocompatibility of Collagen-Based Scaffold Reinforced by Chitin Fibers for Bone Tissue Engineering. J. Biomed. Mater. Res. B Appl. Biomater..

[55] Noh H.K., Lee S.W., Kim J.M., Oh J.E., Kim K.H., Chung C.P., Choi S.C., Park W.H., Min B.M. (2006). Electrospinning of Chitin Nanofibers: Degradation Behavior and Cellular Response to Normal Human Keratinocytes and Fibroblasts. Biomaterials.

[56] Moon H., Choy S., Park Y., Jung Y.M., Koo J.M., Hwang D.S. (2019). Different Molecular Interaction between Collagen and α- or β-Chitin in Mechanically Improved Electrospun Composite. Mar. Drugs.

[57] Madhumathi K., Sudheesh Kumar P.T., Abhilash S., Sreeja V., Tamura H., Manzoor K., Nair S.V., Jayakumar R. (2010). Development of Novel Chitin/Nanosilver Composite Scaffolds for Wound Dressing Applications. J. Mater. Sci. Mater. Med..

[58] Sudheesh Kumar P.T., Srinivasan S., Lakshmanan V.K., Tamura H., Nair S.V., Jayakumar R. (2011). β-Chitin Hydrogel/Nano Hydroxyapatite Composite Scaffolds for Tissue Engineering Applications. Carbohydr. Polym..

[59] Kumar P.T.S., Abhilash S., Manzoor K., Nair S.V., Tamura H., Jayakumar R. (2010). Preparation and Characterization of Novel β-Chitin/Nanosilver Composite Scaffolds for Wound Dressing Applications. Carbohydr. Polym..

[60] Xing F., Chi Z., Yang R., Xu D., Cui J., Huang Y., Zhou C., Liu C. (2021). Chitin-Hydroxyapatite-Collagen Composite Scaffolds for Bone Regeneration. Int. J. Biol. Macromol..

[61] Chang C., Peng N., He M., Teramoto Y., Nishio Y., Zhang L. (2013). Fabrication and Properties of Chitin/Hydroxyapatite Hybrid Hydrogels as Scaffold Nano-Materials. Carbohydr. Polym..

[62] Rolandi M., Rolandi R. (2014). Self-Assembled Chitin Nano Fibers and Applications. Adv. Colloid Interface Sci..

[63] Li X., Feng Q., Cui F. (2006). In Vitro Degradation of Porous Nano-Hydroxyapatite/Collagen/PLLA Scaffold Reinforced by Chitin Fibres. Mater. Sci. Eng. C.

[64] Huang Y., Wang Y., Chen L., Zhang L. (2018). Facile Construction of Mechanically Tough Collagen Fibers Reinforced by Chitin Nanofibers as Cell Alignment Templates. J. Mater. Chem. B.

[65] Mincea M., Negrulescu A., Ostafe V. (2012). Preparation, Modification, and Applications of Chitin Nanowhiskers: A Review. Rev. Adv. Mater. Sci..

[66] Muñoz-Núñez C., Fernández-García M., Muñoz-Bonilla A. (2022). Chitin Nanocrystals: Environmentally Friendly Materials for the Development of Bioactive Films. Coatings.

[67] Fan Y., Saito T., Isogai A. (2008). Chitin Nanocrystals Prepared by TEMPO-Mediated Oxidation of α-Chitin. Biomacromolecules.

[68] Youn D.K., No H.K., Prinyawiwatkul W. (2013). Preparation and Characteristics of Squid Pen β-Chitin Prepared under Optimal Deproteinisation and Demineralisation Condition. Int. J. Food Sci. Technol..

[69] Bogdanova O.I., Polyakov D.K., Streltsov D.R., Bakirov A.V., Blackwell J., Chvalun S.N. (2016). Structure of β-Chitin from Berryteuthis Magister and Its Transformation during Whisker Preparation and Polymerization Filling. Carbohydr. Polym..

[70] Yang F.C., Peters R.D., Dies H., Rheinstädter M.C. (2014). Hierarchical, Self-Similar Structure in Native Squid Pen. Soft Matter.

[71] Ianiro A., Giosia M., Fermani S., Samorì C., Barbalinardo M., Valle F., Pellegrini G., Biscarini F., Zerbetto F., Calvaresi M. (2014). Customizing Properties of β-Chitin in Squid Pen (Gladius) by Chemical Treatments. Mar. Drugs.

[72] Donnadio A., Paul G., Barbalinardo M., Ambrogi V., Pettinacci G., Posati T., Bisio C., Vivani R., Nocchetti M. (2023). Immobilization of Alendronate on Zirconium Phosphate Nanoplatelets. Nanomaterials.

[73] Präbst K., Engelhardt H., Ringgeler S., Hübner H. (2017). Basic Colorimetric Proliferation Assays: MTT, WST, and Resazurin. Cell Viability Assays: Methods and Protocols.

[74] Barbalinardo M., Bertacchini J., Bergamini L., Magarò M.S., Ortolani L., Sanson A., Palumbo C., Cavallini M., Gentili D. (2021). Surface Properties Modulate Protein Corona Formation and Determine Cellular Uptake and Cytotoxicity of Silver Nanoparticles. Nanoscale.

[75] Huang J., Zhong Y., Zhang L., Cai J. (2017). Extremely Strong and Transparent Chitin Films: A High-Efficiency, Energy-Saving, and “Green” Route Using an Aqueous KOH/Urea Solution. Adv. Funct. Mater..

[76] Huang Y., Yao M., Zheng X., Liang X., Su X., Zhang Y., Lu A., Zhang L. (2015). Effects of Chitin Whiskers on Physical Properties and Osteoblast Culture of Alginate Based Nanocomposite Hydrogels. Biomacromolecules.

[77] El Harmoudi H., El Gaini L., Daoudi E., Rhazi M., Boughaleb Y., El Mhammedi M., Migalska-Zalas A., Bakasse M. (2014). Removal of 2,4-D from Aqueous Solutions by Adsorption Processes Using Two Biopolymers: Chitin and Chitosan and Their Optical Properties. Opt. Mater..

[78] Li J., Revol J.-F., Marchessault R. (1996). Rheological Properties of Aqueous Suspensions of Chitin Crystallites. J. Colloid Interface Sci..

[79] Prefecture S. (2004). Dyeing Chitin/Cellulose Composite Fibers with Reactive Dyes. Text. Res. J..

[80] Tanabe T., Okitsu N., Tachibana A., Yamauchi K. (2002). Preparation and Characterization of Keratin—Chitosan Composite Film. Biomaterials.

[81] Wang X.H., Li D.P., Wang W.J., Feng Q.L., Cui F.Z., Xu Y.X., Song X.H., Van Der Werf M. (2003). Crosslinked Collagen/Chitosan Matrix for Artificial Livers. Biomaterials.

[82] Fan Y., Saito T., Isogai A. (2008). Preparation of Chitin Nanofibers from Squid Pen β-Chitin by Simple Mechanical Treatment under Acid Conditions. Biomacromolecules.

[83] Montroni D. (2020). Hierarchically Organized Chitin-Based Matrices. Ph.D Thesis.

[84] Vu R., Jin S., Sun P., Haensel D., Nguyen Q.H., Dragan M., Kessenbrock K., Nie Q., Dai X. (2022). Wound Healing in Aged Skin Exhibits Systems-Level Alterations in Cellular Composition and Cell-Cell Communication. Cell Rep..

[85] Chambers E.S., Vukmanovic-Stejic M. (2019). Skin Barrier Immunity and Ageing. Immunology.

