# Supplementary material for: Sustainable 3D Scaffolds Based on β-Chitin and Collagen I for Wound Dressing Applications
Source: Polymers (Basel). 2025 Jan 8;17(2):140. doi: 10.3390/polym17020140 (PMC11769321; doi:10.3390/polym17020140)
Supplement: Supplementary file 1 [file polymers-17-00140-s001.zip › polymers-3369695-supplementary.pdf]

Supporting Information

Sustainable 3D scaffolds based on β-chitin and collagen I for wound dressing applications

*Marianna Barbalinardo, Giuseppe Falini, and Devis Montroni**

Figure S1: Characterization of the β-chitin fibril dispersion.	p. 2
Figure S2: ATR-FTIR analysis of the porous scaffolds obtained.	p. 3
Table S1: FTIR absorption bands of the scaffolds studied.	p. 4
Table S2: Exposed surface, water absorbed, and the total pore volume.	p. 5
Table S3: Compression tests on the wet porous scaffolds.	p. 5
Table S4: Compression tests on the dry porous scaffolds.	p. 5
Figure S3: Compression profiles of the different specimens tested.	p. 6
Figure S4: Fluorescence micrographs of fibroblast after 72 h.	p. 7

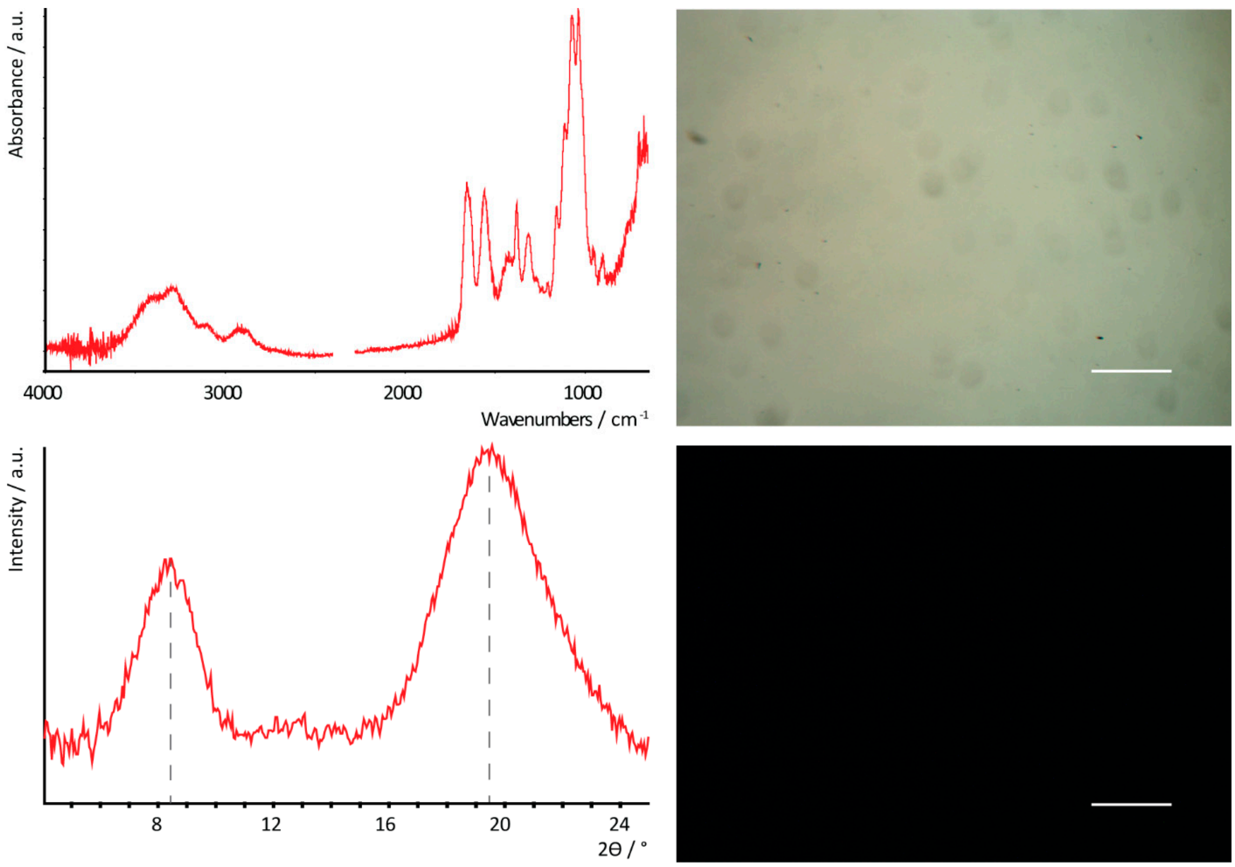

Figure S1:

Characterization of the nano-fibril dispersion obtained. On the left, FTIR (top) and XRD (bottom) of the dispersion dried in an oven. The shape of the C=O stretching absorption band and the position of the X-ray diffraction peaks [8.4° for the (010) reflex and 19.5° for the (100) reflex] confirm the β-chitin polymorph.
In the FTIR, the spectral range corresponding to the CO_2 absorption bands was deleted.

On the right, an optical microscopy image of the dispersion without (top) and with (bottom) cross-polarizers. No microfibers were observed. The spherical objects observable in the optical image are just artifact due to impurities on the microscope lenses that could not be cleaned out and should not be taken into account. Scale bar: 200 µm. Optical microscopy images were collected using a SM-LUX POL microscope equipped with a Moticam 5 5.0 MP camera, a drop of sample was collected and placed on a microscope slide covered with a cover slip.

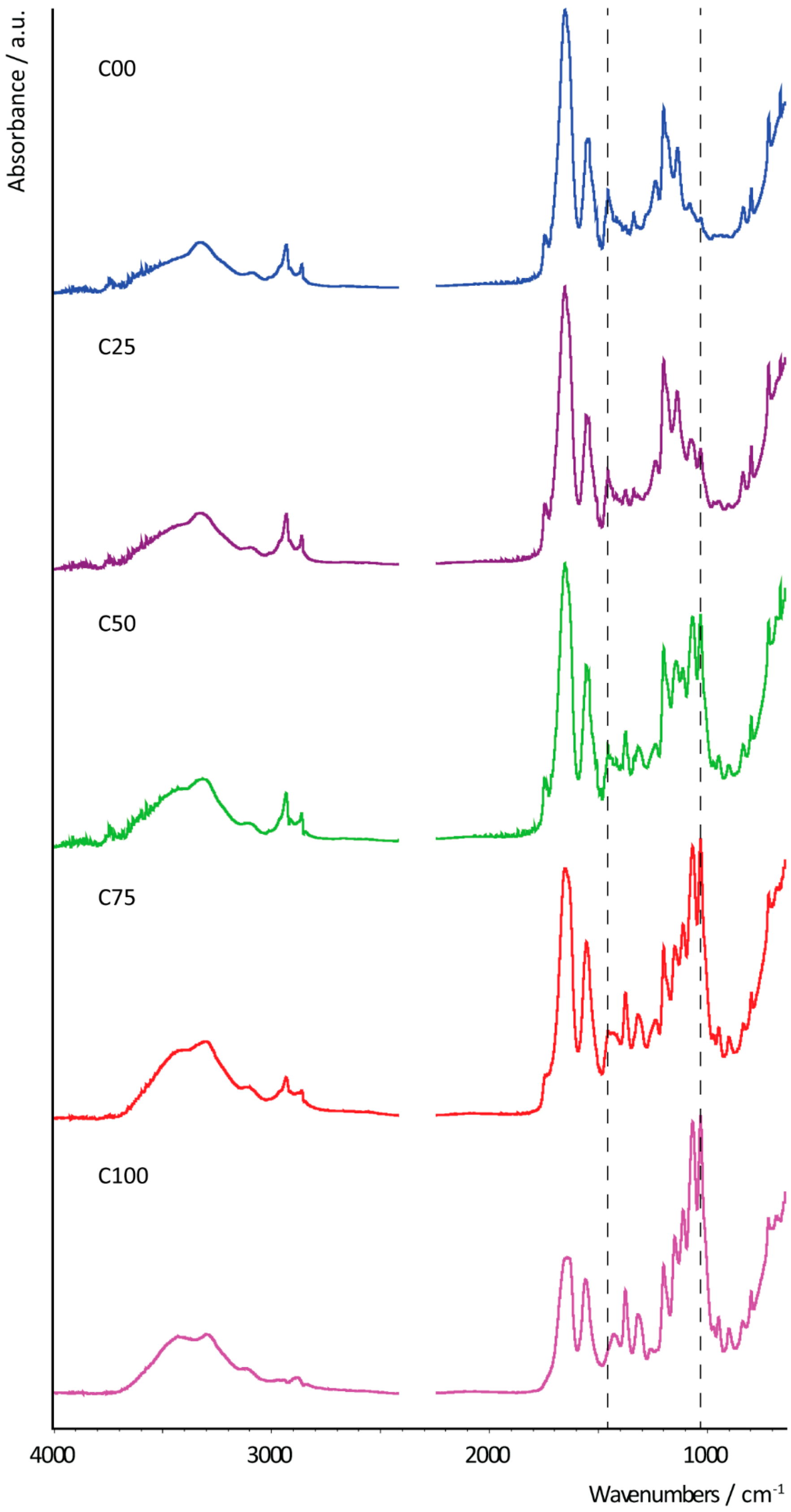

Figure S2: ATR-FTIR spectra of the porous scaffolds obtained. The spectral range corresponding to the CO_2 absorption bands was deleted. The dashed lines indicate two typical absorption bands of chitin (1034 cm^{-1},

C-O stretching) and collagen (1339 cm^{-1}, CH_2 wagging) and show how the difference in relative intensity changes increasing the relative amount of chitin and collagen.

Table S1: FTIR absorption bands (cm^{-1}) for the different scaffolds studied.

Vibration modes	C00	C25	C50	C75	C100
OH stretching	n.d.	3421	3421	3421	3420
NH stretching	3323	3322	3309	3295	3293
NH stretching	3079	3095	3093	3114	3108
CH3 stretching	2927	2927	2927	2928	2938
CH stretching	2855	2855	2856	2856	2878
Amide I of carboxylic acids	1745	1744	1744	n.d.	n.d.
Amide I band	1653	1653	1654	1654	1648
Amide II band	1544	1558	1558	1559	1560
CH_2 bending and CH_3 deformation	1466	1457	1457	1437	1430
CH bending and symmetric CH_3 deformation	n.d.	1375	1376	1377	1377
CH_2 wagging	1339	1339	1319	1319	1320
	n.d.	n.d.	n.d.	n.d.	1261
Amide III	1239	1239	1239	n.d.	n.d.
Amide III	1202	1202	1202	1202	1202
Asymmetric bridge oxygen stretching	1139	1139	1143	1144	1151
Asymmetric in-phase ring stretching mode	n.d.	n.d.	1115	1116	1113
CO stretching	n.d.	1078	1070	1070	1070
CO stretching	n.d.	1034	1034	1034	1034
CH3 wagging	n.d.	n.d.	950	952	952
Ring stretching	n.d.	n.d.	905	905	904
	837	840	840	841	841
	801	801	801	801	802

Table S2: Exposed surface, water absorbed, and the total percentage of volume in the scaffold occupied by pores are reported for each scaffold synthetized. (*) A cylindrical geometry could not be assumed for this sample.

	Water absorbed / wt.%			Pores / vol.%			Exposed surface / $m^2 \cdot g^{-1}$		
C00	3300	±	100		*		4.3	±	0.1
C25	6300	±	200	73	±	6	5.61	±	0.07
C50	6700	±	200	71	±	4	6.3	±	0.1
C75	7000	±	300	80	±	3	7.25	±	0.05
C100	7100	±	700	83	±	7	10.24	±	0.07

Table S3: Compression tests on the wet porous scaffolds. Because of the geometry of the scaffold, it was not possible to obtain reliable data on the C00 scaffold.

	Young modulus / Pa			Tenacity / kPa			Densification / %			Densification / kPa		
C25	17	±	2	7.0	±	0.5	42	±	5	1.4	±	0.1
C50	14	±	2	6.7	±	0.4	50	±	5	1.5	±	0.1
C75	20.1	±	0.7	8.8	±	0.3	61	±	5	2.6	±	0.6
C100	24	±	2	9.7	±	0.8	58	±	10	2.8	±	0.8

Figure S3: Compression profiles of the different specimens tested.

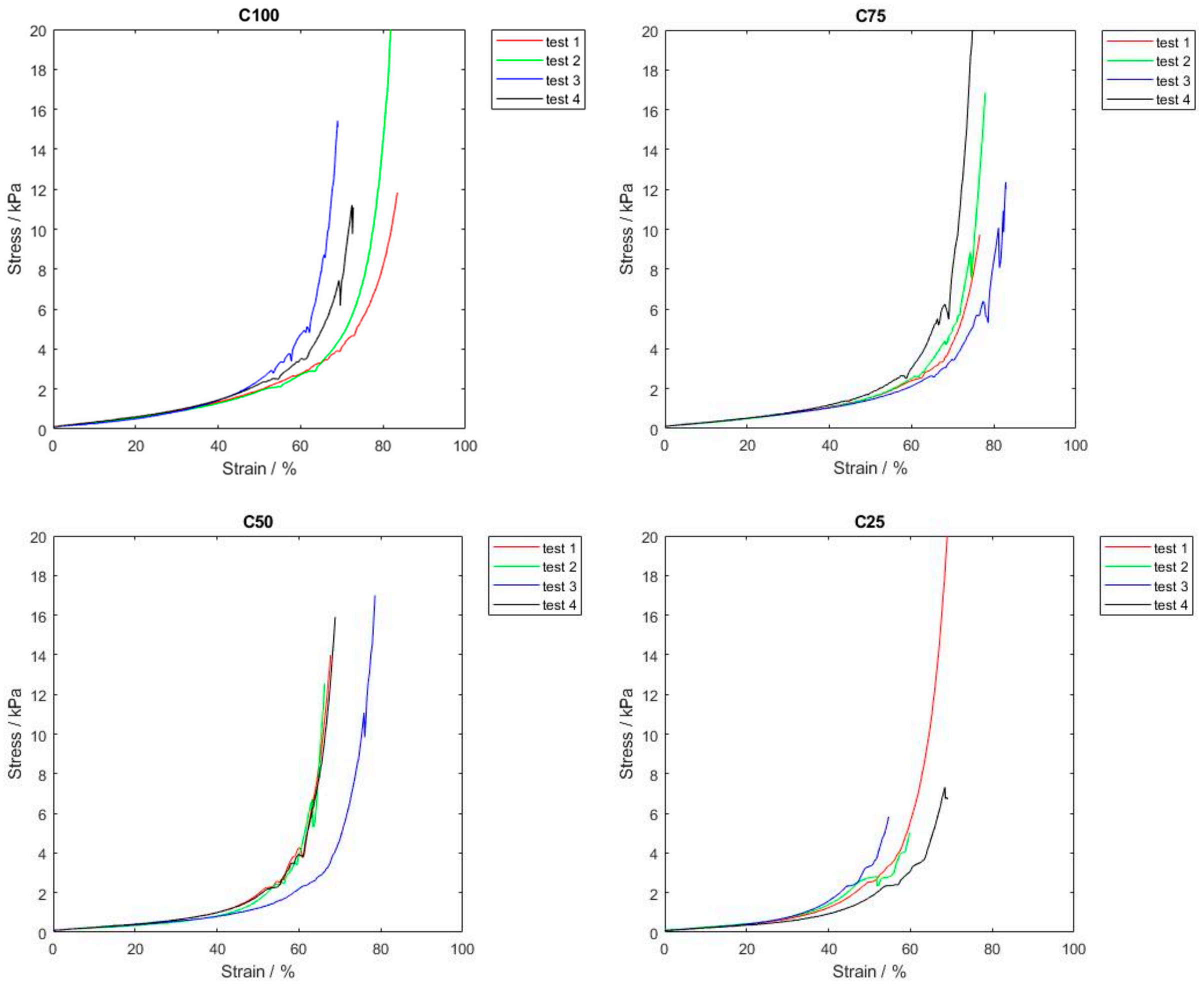

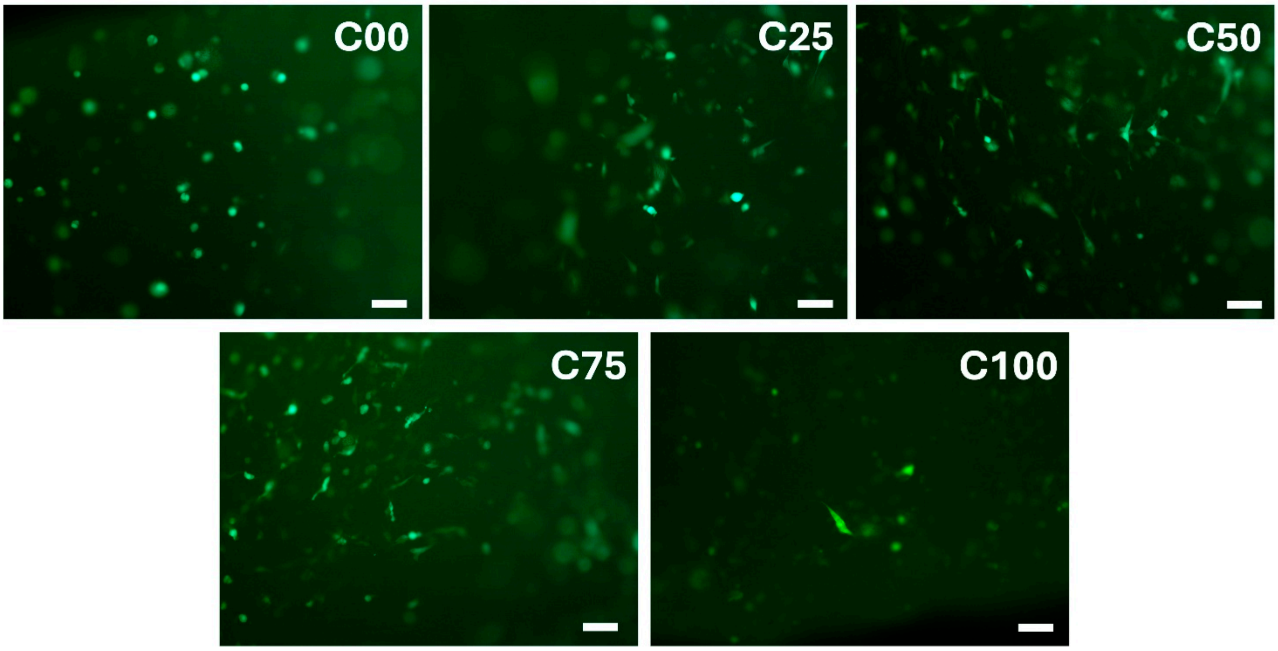

Figure S4: Fluorescence micrographs of fibroblast labeled with green florescence protein after 72 h on the scaffolds (scale bar: 100 µm).
